# Increased serum homocysteine in first episode and drug-naïve individuals with schizophrenia: sex differences and correlations with clinical symptoms

**DOI:** 10.1186/s12888-022-04416-x

**Published:** 2022-12-03

**Authors:** Xu Yang, Haidong Yang, Na Li, Chunyu Li, Weiye Liang, Xiaobin Zhang

**Affiliations:** 1 Department of Psychiatry, Beijing Hui Long Guan Hospital, Peking University Hui Long Guan Clinical Medical School, Beijing, 100096 People’s Republic of China; 2 Department of Psychiatry, The Fourth People’s Hospital of Lianyungang, The Affiliated KangDa College of Nanjing Medical University, Lianyungang, 222003 People’s Republic of China; 3 Department of Pathology, Beijing Kingmed Clinical Laboratory, Affiliated Peking University, Beijing, 100015 People’s Republic of China; 4 Clinical Laboratory, Beijing Hui Long Guan Hospital, Peking University Hui Long Guan Clinical Medical School, Beijing, 100096 People’s Republic of China; 5 Institute of Mental Health, Suzhou Psychiatric Hospital, The Affiliated Guangji Hospital of Soochow University, Suzhou, 215137 People’s Republic of China

**Keywords:** Schizophrenia, Homocysteine, Sex difference, Pathophysiology

## Abstract

**Background:**

Accumulating evidence shows that homocysteine (Hcy) is implicated in the pathophysiology of schizophrenia, and plays an important role in clinical characteristics. This study evaluated the relationships between Hcy levels and clinical features in first-episode, Chinese Han, drug-naïve (FEDN) patients with schizophrenia.

**Methods:**

FEDN individuals (119 with schizophrenia and 81 healthy controls matched for age, sex, education, and body mass index (BMI)) were enrolled. The serum Hcy levels were determined by enzyme cycle assay experiments. Severities of clinical symptoms were rated on the Positive and Negative Syndrome Scale (PANSS).

**Results:**

FEDN individuals with schizophrenia had higher Hcy levels compared with healthy controls (F = 46.865, *P* < 0.001). Correlation analysis and multiple stepwise regression analyses showed that serum Hcy levels in FEDN schizophrenia individuals were positively correlated with PANSS general psychopathology subscale (*r* = 0.294, *P* = 0.001) and PANSS total score (*r* = 0.273, *P* = 0.003). No significant association was found between Hcy and age, BMI, PANSS positive subscale, and the PANSS negative subscale (all, *P* > 0.05). Male individuals had significantly higher serum Hcy levels than female individuals (F = 7.717, *P* = 0.006) after controlling for confounding factors (F = 0.759, *P* = 0.011).

**Conclusions:**

Serum Hcy levels were increased in FEDN individuals with schizophrenia, and Hcy levels may be involved in pathophysiological mechanisms. Sex differences in Hcy levels were observed, with higher levels in male FEDN individuals compared to females.

## Introduction

Schizophrenia has an insidious onset, and is a chronic and highly disabling mental disorder, which affects approximately 1% of the worldwide population [1]. The manifestations of schizophrenia are heterogeneous in term of positive symptoms, negative symptoms, and cognitive impairments, which mostly occur in early adulthood [2, 3]. The availability of atypical antipsychotics has alleviated or mitigated some symptoms of schizophrenia, but characteristics of the disorder still constitute a major social burden [4, 5]. However, the pathophysiological mechanism of schizophrenia still remains multifactorial and unclear.

Homocysteine (Hcy) is a non-protein and sulfur-containing amino acid originating from the essential amino acid, methionine, which is derived from dietary proteins and involved in many pathophysiological processes [6]. Hcy is a pro-oxidant, which contributes to oxidative stress in nerve cells [7]. Previous studies have reported that Hcy interacted with N-methyl-D-aspartate receptors or participated in oxidative stress, nitrosative stress, the transsulfuration pathways, pro-inflammatory states, mitochondrial dysfunction, and DNA methylation [8–10]. A mouse model has also revealed a close relationship between hyperhomocysteinemia and inflammatory cytokines activated via NF-κB and microglial cells [11]. These studies partially explain the mechanisms involved in Hcy abnormalities leading to schizophrenia. A Mendelian randomization study reported that increased plasma Hcy levels increased the risk of schizophrenia and bipolar disorders [12]. A systematic review and meta-analysis reported that levels of Hcy were higher in first-episode psychosis compared to controls, suggesting that an imbalance of antioxidant status may be relevant to first-episode psychosis [13]. In addition, the relationships between abnormal Hcy levels and schizophrenia and clinical symptoms have been reported in different countries such as United States [14], Tunisia [15], Republic of Korea [16], Turkey [17], and India [18]; however, the results are controversial and even some negative results have been reported [19, 20].

Moreover, stress, poor lifestyle, age, catabolic dysregulation, and genetics can lead to abnormalities in Hcy, while abnormal Hcy levels may be considered as an etiological or risk factor in neurodegenerative diseases such as dementias, Alzheimer’s disease, Parkinson’s disease, cardiovascular diseases, and metabolic disease [21–23], but individuals in northern Italy with severe mental illness may not be at higher risk of cardiovascular disease than the general population, especially in the relatively wealthy areas and with traditional healthy dietary habits such as the Mediterranean diet [24]. A recent Mendelian Randomization study reported that in the general population, higher Hcy concentration was a risk factor for metabolic syndrome, but not for body mass index (BMI) [25], while another study reported that higher Hcy levels correlated with lower BMIs in schizophrenia patients [26]. In addition, several studies have reported that sex may also be a factor affecting Hcy in the general population [27], in bipolar disorders [28], and even in rat animal models [29]. First-episode drug-naïve (FEDN) individuals with schizophrenia are the optimal population to evaluate the relationships between Hcy and schizophrenia and clinical features, because of the absence of confounding factors such as medication, BMI, diet, and the stage of illness.

The present study therefore aimed to assess Hcy levels and its associations with clinical features in FEDN schizophrenia individuals of Chinese Han ethnicity. We hypothesized that Hcy levels may be altered and may be correlated with schizophrenia clinical features. The results showed (1) whether serum Hcy levels differed between FEDN individuals with schizophrenia and healthy controls and (2) whether there were associations between serum Hcy levels, sex, and BMI, and the severities of clinical symptoms.

## Methods

### Subjects and clinical assessments

FEDN schizophrenia individuals were enrolled at Beijing Hui-Long-Guan Hospital. The inclusion criteria included (1) age of 18—45 years, (2) Han Chinese ethnicity, (3) meeting the criteria of schizophrenia according to the Structured Clinical Interview of the Diagnostic and Statistical Manual-IV by two psychiatrists, and (4) patients were never treated with antipsychotics. Their demographic data included sex, age, years of education, smoking, BMI, duration of illness, and age at onset, using a questionnaire. The Positive and Negative Syndrome Scale (PANSS) [30] was used by two experienced psychiatrists to assess the psychopathological status of FEDN schizophrenia individuals. The PANSS score of interrater correlation was 0.8.

Healthy controls were enrolled after responding to advertisements in Beijing. They were matched for sex, age, years of education, and BMI. Individuals were also evaluated for mental status using Axis I disorders. Candidates who met psychiatric disorders or a family history for psychiatric disorders were excluded.

The health status of each participant was identified by physical examinations and laboratory tests. Individuals with the following conditions were excluded: mental retardation, epilepsy, traumatic or chronic brain injury, diabetes, thyroid diseases, infections, cardiovascular and cerebrovascular diseases, pregnancy, and drug or alcohol abuse/dependence.

All subjects or their guardians gave informed written consent, and the research protocol was approved by the Ethics and Research Committee of Beijing Hui-Long-Guan Hospital.

### Hcy determination

WE used an EDTA tube to collect venous blood samples from patients and healthy controls between 08:00 and 09:00 after overnight fasting. The collected blood samples were immediately centrifuged (3000 rpm) for collection of serum for Hcy concentrations, which were determined by enzyme cycle assay experiments using commercially available kit instructions (Beijing Leadman Biotechnology, Beijing, China). Sample quality was observed before detection to exclude hemolysis, celiac blood, etc. All blood samples were measured by standard blood biochemistry assays on a AU5800 automated biochemical analyzer (Beckman Coulter, Tokyo, Japan). The parameters were set before the assay: R1 reagent, sample and R2 reagent were added at 0, 18 and 198 s, respectively, and the first measurement time point was 396 s, the second measurement time point was 486 s, the primary wavelength was 340 nm and the secondary wavelength was 700 nm. The same investigator who was blinded to the clinical status tested all samples.

### Statistical analysis

All data were analyzed using the Statistical Package for Social Sciences (SPSS), version 19.0 (SPSS, Chicago, IL, USA). If data were nonparametric according to the Kolmogorov–Smirnov test, they were transformed into normally distributed data using a logarithm. The transformed values were used for comparisons, while the original data were used for descriptions and were expressed as the median (25th and 75th quartiles). As Hcy levels was not normally distributed, we log-transformed the Hcy values. Continuous variables between groups were analyzed using analysis of variance, and expressed as mean ± standard deviation, and categorical variables were analyzed using the chi-squared test. Analysis of covariance was used to analyze potential confounding variables including sex, age, years of education, and BMI. Pearson’s correlation coefficients were used to evaluate the associations between variables. The Bonferroni correction was used to control for multiple tests. Multiple stepwise regression analysis was used to assess the relationship of Hcy levels with demographic and clinical characteristics. *P* < 0.05 was considered statistically significant.

## Results

### Sociodemographic characteristics

A total of 119 FEDN schizophrenia subjects were enrolled including 60 males and 59 females, with average age of 27.73 ± 7.54 years, mean years of education of 12.23 ± 2.00 years, mean duration of untreated illness of 1.99 ± 1.83 years, average age at onset of 25.75 ± 6.56 years, and BMI of 24.62 ± 2.96 kg/m^2^. A total of 81 healthy controls were enrolled, including 42 males and 39 females. Table 1 shows the demographics and clinical profiles of FEDN schizophrenia individuals and healthy controls. There was no significant difference in sex, age, years of education, smoking, and BMI (all *P* > 0.05).

**Table 1:** Demographics and clinical characteristics of FEDN patients with schizophrenia and healthy controls

	FEDN (*n* = 119)	HC (*n* = 81)	F/χ^2^	*P*
Age (years)^a^	27.73 ± 7.54	25.95 ± 6.56	2.979	0.086
Sex (M/F)	60/59	42/39	0.040	0.842
Education (years)^a^	12.23 ± 2.00	12.57 ± 2.31	1.235	0.268
Smoking (Yes/No)	25/94	13/68	0.770	0.380
BMI (kg/m^2^)^a^	24.62 ± 2.96	24.30 ± 2.39	0.609	0.436
Duration of untreated illness (years)^a^	1.99 ± 1.83			
Age at onset (years)^a^	25.75 ± 6.56			
PANSS positive subscore^a^	27.91 ± 7.33			
PANSS negative subscore^a^	16.09 ± 7.58			
PANSS general subscore^a^	25.18 ± 9.29			
PANSS total score^a^	69.18 ± 14.81			
Hcy (μmol/L)^b^	19.80 (15.51, 16.40)	13.01 (11.65, 16.55)	46.865	** < 0.001**

*FEDN* first-episode drug-naïve, *HC* healthy control, *BMI* body mass index, *PANSS* Positive and Negative Syndrome Scale, *Hcy* homocysteine

^a^The data were expressed as the mean ± standard deviation

^b^The data were expressed as the median (25th quartile and 75th quartile). Significant difference (*P* < 0.05) is indicated in bold

### Serum Hcy concentrations in FEDN individuals and healthy controls

**Fig. 1:**
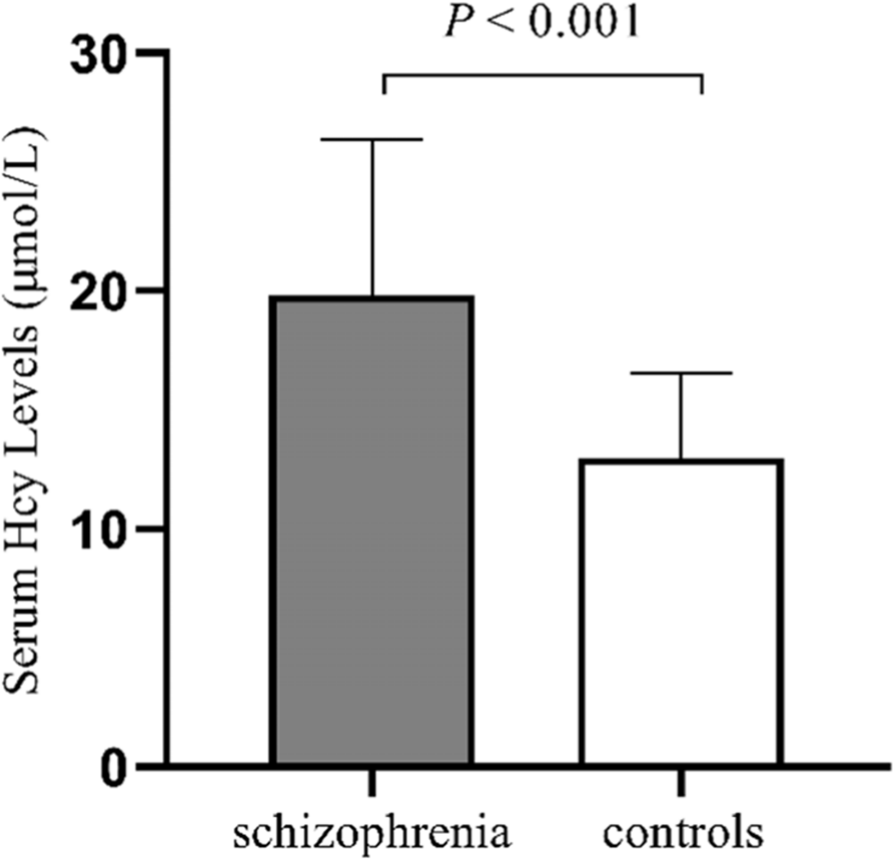
Serum levels of Hcy in FEDN patients with schizophrenia and in healthy controls

Because the serum Hcy concentrations were nonparametrically distributed both in FEDN individuals and healthy controls according to the Kolmogorov–Smirnov test (*P* < 0.05), we first transformed the data into normal distributions using logarithmic transformation. The log-transformed values for individuals with schizophrenia and controls groups were 1.32 ± 0.21 vs 1.15 ± 0.13 μmol/L, and analysis of variance showed that serum Hcy concentrations were significantly higher in FEDN individuals with schizophrenia than in healthy controls (*P* < 0.001) (Table 1, Fig. 1). Analysis of variance also showed that there was significant difference after controlling for sex, age, years of education, smoking, and BMI (F = 44.49, *P* < 0.001).

### Associations between serum Hcy concentrations and clinical symptoms

**Fig. 2:**
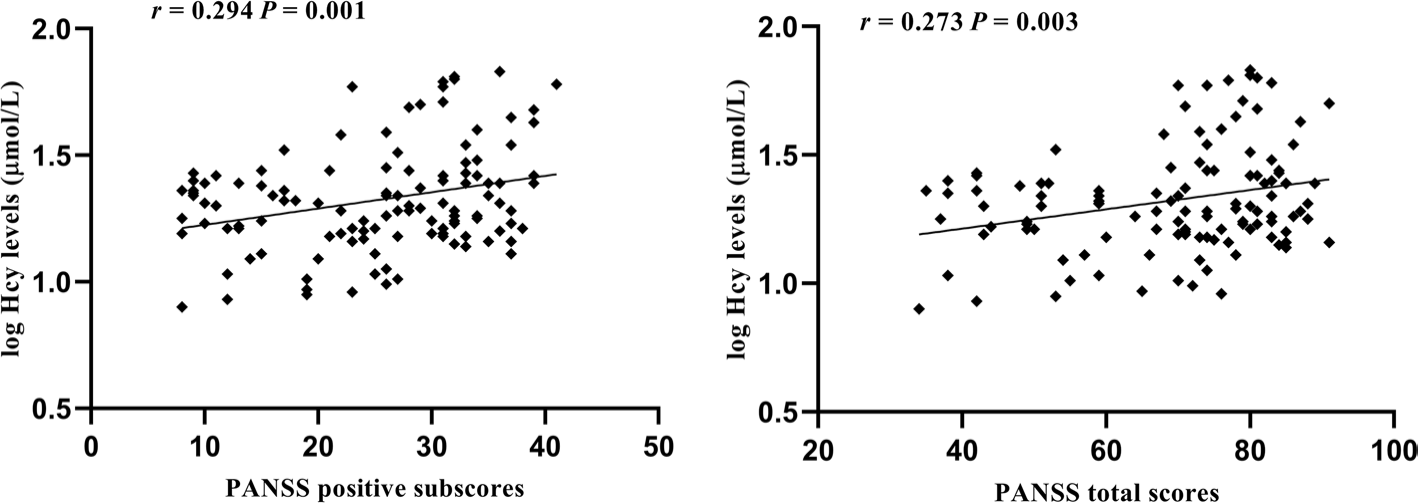
Correlations between Hcy levels and PANSS positive subscores and total scores

Pearson’s correlation analysis showed that serum Hcy concentration was positively associated with PANSS general subscore (*r* = 0.294, df = 119, *P* = 0.001) and PANSS total score (*r* = 0.273, df = 119, *P* = 0.003) in FEDN schizophrenia individuals (Fig. 2). There were also significant differences after Bonferroni corrections (all *P* < 0.05), but no significant association between levels of Hcy and the PANSS positive subscale, PANSS negative subscale, and BMI (all *P* > 0.05).

Multiple stepwise regression showed that sex (beta = 0.694, t = 10.625, *P* < 0.001), duration of illness (beta = -0.168, t = -2.636, *P* = 0.010), and Hcy (beta = 0.144, t = 2.200, *P* = 0.030) were influencing factors for the PANSS general psychopathology subscale (*R*^2^ = 0.544), and sex (beta = 0.554, t = 7.335, *P* < 0.001), duration of illness (beta = -0.228, t = -3.087, *P* = 0.003), and Hcy (beta = 0.166, t = 2.197, *P* = 0.030) were correlated with PANSS total scores (*R*^2^ = 0.390) after controlling for age, years of education, age at onset, smoking, and BMI.

### Sex differences in Hcy levels with clinical symptoms

Table 2 shows that serum Hcy levels, PANSS positive symptoms, negative symptoms, general psychopathology, and PANSS total scores were significantly different between male and female patients (all *P* < 0.05) except for age, years of education, BMI, duration of illness, and age at onset (all *P* > 0.05).

**Table 2:** Characteristics of male and female FEDN patients with schizophrenia

	Male	Female	F	P
Age (years)^a^	28.62 ± 8.05	26.83 ± 6.95	1.677	0.198
Education (years)^a^	12.18 ± 2.00	12.27 ± 2.02	0.057	0.812
BMI (kg/m^2^)^a^	24.52 ± 3.48	24.71 ± 2.34	0.115	0.735
Duration of illness (years)^a^	2.22 ± 2.18	1.75 ± 1.35	1.999	0.160
Age at onset (years)^a^	26.40 ± 6.72	25.08 ± 6.37	1.198	0.276
PANSS positive subscale^a^	25.67 ± 7.81	30.19 ± 6.06	12.421	**0.001**
PANSS negative subscale^a^	20.12 ± 7.65	12.00 ± 4.85	47.550	** < 0.001**
PANSS general subscale^a^	31.68 ± 6.21	18.58 ± 6.95	117.764	** < 0.001**
PANSS total score^a^	77.47 ± 9.60	60.76 ± 14.48	55.211	** < 0.001**
Hcy (μmol/L)^b^	21.95 (16.25, 29.03)	19.10 (14.90, 23.80)	7.717	**0.006**

*BMI* body mass index, *PANSS* Positive and Negative Syndrome Scale, *Hcy* homocysteine

^a^The data are expressed as the mean ± standard deviation

^b^ The data are expressed as the median (25th quartile and 75th quartile). Significant differences (*P* < 0.05) are indicated in bold

After adjusting for age, years of education, smoking, BMI, duration of illness, and age at onset, we found using analysis of variance that Hcy levels were significantly higher in male FEND schizophrenia patients than in females (F = 6.759, *P* = 0.011). No significant difference in Hcy level was found between male and female healthy controls (*P* > 0.05).

## Discussion

IN the present study, we found that (1) FEDN individuals with schizophrenia had higher serum Hcy levels than healthy controls, (2) serum concentrations of Hcy were positively correlated with the PANSS general psychopathology subscale and PANSS total scores for patients, and (3) male patients had significantly higher Hcy levels than female patients. To the best of our knowledge, few studies have reported the sex difference in serum Hcy levels in FEDN individuals with schizophrenia of Chinese ethnicity [31, 32].

The present study showed that serum levels of Hcy were significantly increased in FEDN schizophrenia individuals, when compared to healthy controls, which is consistent with other reports [26, 33]. A cross-sectional study reported that Hcy levels were increased in schizophrenia, when compared with bipolar disorder patients [34]. Another study reported that common polygenic variants, such as methylenetetrahydrofolate reductase (MTHFR) C677T polymorphism, correlated with plasma total Hcy levels, which had a cumulative effect on schizophrenia and may be a risk factor for this disorder [35]. There is also increasing evidence that Hcy is directly or indirectly involved in multiple pathways in pathophysiological mechanisms of schizophrenia. Methylation pathway defects (including catecholamine methylation) due to folate and cobalamin deficiencies, and impaired re-methylation of methionine by Hcy, can cause hyperhomocysteinemia [36]. Another impaired pathway is transsulfuration pathway, which is a metabolic pathway from Hcy to L-cysteine to glutathione (GSH), resulting in reduced GSH levels and oxidative phosphorylation [37]. In addition, Hcy induces immune responses, and in a mouse model, elevated Hcy activates nuclear translocation of transcription factor NF-κB and increased expressions of interleukins-1β and tumor necrosis factor-α [11]. However, the exact etiology and pathways of Hcy in schizophrenia remain ambiguous and warrant further study.

Another finding of the study was that serum Hcy levels were positively correlated with psychopathological symptoms of FEDN schizophrenia individuals. Similar results have been reported in several previous studies. Trześniowska-Drukała et al. [38] reported that increased Hcy concentrations resulted in worse cognitive functions and higher PANSS scores in patients with schizophrenia, suggesting that Hcy blood levels were related to the severity of schizophrenia. Song et al. [39] reported that increased serum Hcy levels positively correlated with PANSS total scores in FEDN schizophrenia patients after controlling for influencing factors. Petronijević et al. [40] reported that plasma Hcy levels were positively correlated with PANSS negative subscores both in the exacerbation and remission phases of young male schizophrenia patients. Gao et al. [41] reported that MTHFR C677T polymorphism was correlated with PANSS negative symptoms and cognitive deficits in chronic schizophrenia patients. Furthermore, models fed Hcy were found to have significantly impaired cognitive functions during the reversal phase of the Morris water maze test [42]. These findings suggested that increased Hcy levels were closely related to the severity of clinical symptoms and cognitive symptoms of schizophrenia in different phases of pathology. The exact pathological mechanisms underlying the correlation between Hcy and the severity of clinical symptoms are not known. However, a review reported that Hcy may regulate dopamine, serotonin, acetylcholine, glutamate function, and the interaction between Hcy and oxidative stress and neuronal apoptosis, and that aberrant DAN methylation were related to schizophrenia [43]. Together, these results suggested that elevated Hcy may play a role in the disease process of schizophrenia, possibly as a consequence of pathological processes involved in schizophrenia or as a causative factor of schizophrenia, so the relationship between elevated Hcy levels and the clinical symptoms of schizophrenia needs further study.

Previous studies have proposed there are several factors influencing Hcy levels such as age, sex, BMI, smoking, coffee drinking, poor nutrition, vitamin intake, folate, and ultraviolet radiation [44, 45]. Previous studies have reported differences in Hcy levels between male and female schizophrenia patients, although the results have been inconsistent. Meta-analyses suggested that plasma total Hcy levels were higher in both male and female schizophrenia patients [46]. A cross sectional study also reported no significant disparity in hyperhomocysteinemia between male and female schizophrenia patients [34]. In the present study, we found that the serum Hcy levels were higher in male patients with FEDN schizophrenia, when compared with female patients, which was consistent with the findings of an earlier study [42]. Another study reported that male sex and older age were risk factors for developing hyperhomocysteinemia in schizophrenia patients [31]. These inconsistencies in the results may be due to different antipsychotic medications, methods of Hcy measurement, different phases of the disease, nutrient status, and ethnicity. Notably, sex differences in Hcy levels were found in patients with schizophrenia, and also in patients with bipolar disorders [47], and Alzheimer’s dementia [48]. However, the exact etiological mechanism underlying the discrepancy in Hcy levels between males and females remains unclear. A large cross-sectional study in the general population reported that the ratio of increased plasma Hcy concentrations, defined as abnormal Hcy results at above 15 μmol/L, were higher in males than in females [49]. The study reported that a possible explanation for the differences in Hcy between males and females was that Hcy metabolism was different, which involved the different pathways of trans-sulfuration and methylation. A review stated that high dose testosterone administration increased total Hcy levels in female-to-male transsexuals [50]. Two other studies reported that hormone treatment, oral ethinyl estradiol and transdermal 17beta estradiol treatments for male-to-female transsexuals reduced Hcy levels [51, 52]. Moreover, one study reported a significant sex disparity in Hcy levels for MTHFR C677T polymorphism [46]. Consequently, we postulate that hormone levels and genetics may be causative factors contributing to sex differences in Hcy levels.

In the present study, we found that severities of clinical symptoms were significantly different between male and female FEDN individuals with schizophrenia, which were consistent with the results of other studies [53, 54]. However, some discrepant results were reported due to different stages of illness, duration of illness, representation of research subjects, and administration of medications. In addition, there was no significant relationship of Hcy concentrations for BMI in our study. Although the results are the same as previously reported [55], there are nevertheless many reports of varying results, suggesting that Hcy concentrations correlated with BMI in patients with schizophrenia [26, 56]. The Hcy levels were closely associated with elderly males, obesity, uric acid content, glycolipid metabolism, and metabolic syndromes [31, 48, 57], whereas our study subjects were on average young and unmedicated, mitigating the effects of confounding factors on Hcy levels, so the results may be more accurate.

There were several limitations in this study. First, based on a cross-sectional study, a causal interaction between Hcy and clinical profile could not be determined. Second, the study subjects were recruited from only one region, and it is possible that different dietary habits and nutrition may have had an impact on the results, so future studies will need to be conducted in a more general area. Third, we did not collect markers related to B vitamins, folate, and glycolipid metabolism, which previous studies suggested may have had an effect on Hcy levels. Fourth, in our study, the number of control sample was less than the patient sample, and will need to be matched in further studies.

In summary, our findings confirmed that serum Hcy levels were higher in FEDN individuals with schizophrenia, when compared with healthy controls, and that higher Hcy levels were correlated with severe clinical symptoms. Importantly, male FEDN schizophrenia individuals had higher Hcy levels than female individuals. Considering the limitations, the present study preliminarily suggested that Hcy played an important role in the pathophysiology of schizophrenia, which requires further validation in a longitudinal study.

## Data Availability

The datasets used and analyzed during the current study are available from the corresponding author on reasonable request.
